# JC virus-induced progressive multifocal leukoencephalopathy in a presumably healthy patient

**DOI:** 10.1186/s12883-022-03004-6

**Published:** 2022-12-09

**Authors:** Xiang Wang, Jinxiu Chen, Jing Gong, Ying Wu, Xiang-hao Liu

**Affiliations:** 1 Department of Neurosurgery, West China Hospital of Sichuan University, Chengdu, 610041 China; 2 Department of Radiology, Sichuan Cancer Center, School of Medicine, Sichuan Cancer Hospital & Institute, University of Electronic Sciences and Technology of China, Chengdu, China; 3 Department of Pathology, West China Hospital of Sichuan University, Chengdu, China; 4 Department of Neurology, West China Hospital of Sichuan University, Chengdu, China

**Keywords:** Progressive multifocal leukoencephalopathy, JC virus, Immunocompetence, Mirtazapine, Mefloquine

## Abstract

**Background:**

JC virus (JCV) is common among healthy individuals and remains latent but may be reactivated under immunosuppressive conditions, resulting in progressive multifocal leukoencephalopathy (PML). Here, we present a rare case of PML caused by JC virus infection in a previously healthy and immunocompetent patient.

**Case presentation:**

A 67-year-old female without any disease history was admitted after presenting with rapidly progressive dementia. The preoperative diagnosis was progressive multifocal leukoencephalopathy, and corticosteroid treatment did not improve the symptoms. Brain lesions were surgically sampled, and JCV infection was confirmed by high-throughput DNA gene detection. This patient received a combined treatment of mirtazapine, mefloquine, and traditional Chinese herbs, and had stabilization of the disease on followed-up.

**Conclusions:**

Although it is a rare, this case demonstrates that JC virus infection within the brain occurs in apparently healthy people. This case may increase our understanding of virus infection when facing the coronavirus epidemic in recent years, considering that similar medications were used.

**Graphical Abstract:**

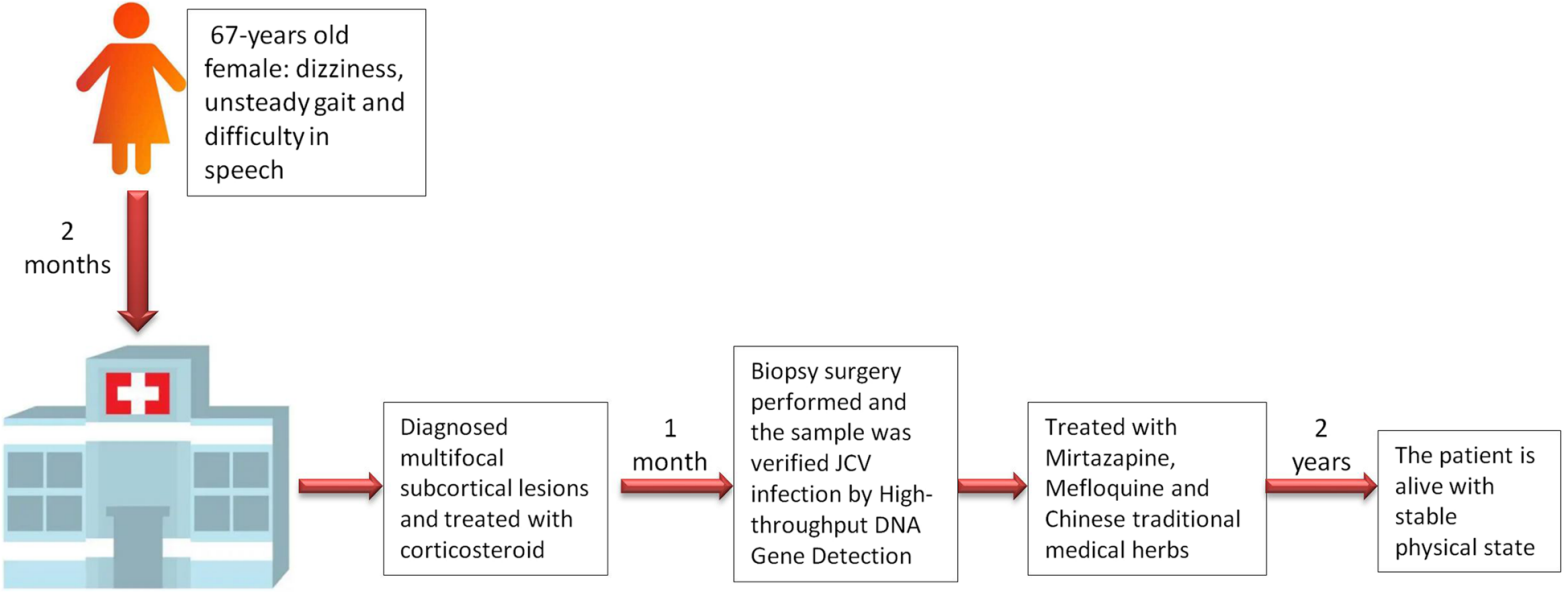

## Background

Progressive multifocal leukoencephalopathy (PML) is a fatal and destructive demyelinating disease of the central nervous system with subacute progression and poor prognosis. PML, when caused by the JC virus (JCV) infection within the brain, almost exclusively occurs in immunocompromised patients, such as HIV/AIDS, organ transplant recipients, and those receiving immunosuppressant therapies. JCV is common among healthy individuals and remains latent, with approximately 70%~80% seropositivity in adults [1]. However, it may be reactivated under immunosuppressive conditions, resulting in PML. Although there are few reported cases [2], we present a rare case of PML in a healthy and immunocompetent patient who received medical treatment and followed-up.

## Case presentation

A 67-year-old female was admitted to our hospital in September 2019 with progressing dizziness, unsteady gait, and speech difficulty. The patient was previously healthy without history of tumor, HIV, or organ transplantation, and without the usage of immunosuppressants or monoclonal antibodies. Any autoimmune diseases/immunodeficiency were not known before. Physical examination showed a serious dysfunction of cognition, with a decrease in emotion, memory, and intelligence. Barthel index for activities of daily living (ADL) on admission scored 35 points, indicating a severe dependency. Infectious sources, including blood, urine, and CSF were negative. T cell subsets in peripheral blood, including CD3, CD4 and CD8, were detected normal. And the ratio of CD4/CD8 was 1.26. But some parameters, such as angiotensin converting enzyme (ACE), neopterin, interleukin (IL-)-2-receptor-antibodies, lysozyme, oligoclonal bands, and JCV, were not analyzed in CSF and serum. Contrast-enhanced chest and abdominal computed tomography showed no mass lesions or swollen lymph nodes.

**Fig. 1:**
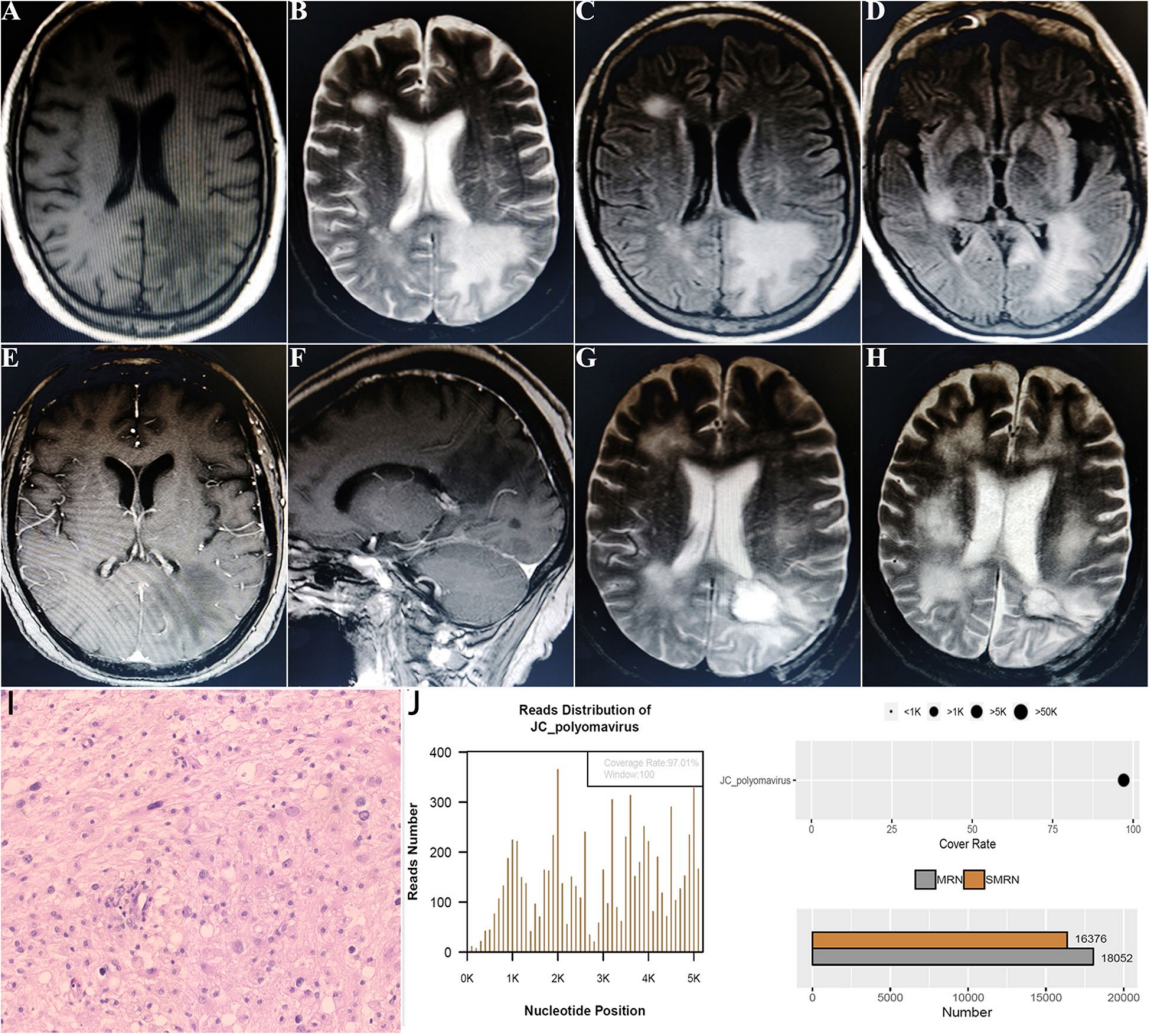
MR imaging findings, histopathological specimens and gene detection of JCV-induced PML. **a**-**d** Multiple lesions in both hemispheres with hypointensity on T1 imaging and hyperintensity on T2 or FLAIR imaging; **e**, **f** Axial and sagittal contrast MR imaging revealed a large massive lesion located in the left parietal lobe with slight dotted enhancement. **g**, **h** Immediate and 24-month postoperative MR imaging. **I** Histopathological specimens revealed some infected cells with enlarged or bizarre-shaped nuclei, with a background of infiltration of plasma cells, lymphoid cells, and abundant macrophages. **J** A “High-throughput DNA Gene Detection of Pathogenic Microorganisms” confirmed the infection of JC virus

Serial MRI on admission revealed multifocal subcortical lesions in both hemispheres, which were hypointense on T1 and hyperintense on T2 or FLAIR imaging with a slight dotted enhancement in the center (Fig. 1A-F). Almost all lesions grew around the lateral ventricle, and the largest lesion was located on the left parietal lobe. Intravenous corticosteroids were administered initially, however the mental status of the patient worsened. Surgical biopsy was performed, and a large mass of tissue on the left parietal lobe was obtained for further pathological and genomic detection (Fig. 1G). Lymphoid cell infiltration was observed in the frozen sample. The patient was stuporous after surgery, similar to her mental status prior to surgery.

Histopathological specimens revealed infiltration of plasma cells, lymphoid cells, and abundant macrophages. Some cells with enlarged or bizarre-shaped nuclei were present, suggesting infected cells (Fig. 1I). Immunohistochemistry (IHC) analysis showed GFAP (+), Olig2 (+), ATRX (+), EMA (-), NeuN (-), and S-100 (+) in glial cells; P53 (+), Ki67 (+) in cells with bizarre-shaped nuclei; CD163 (+), CD68 (+), and CD1a (-) in macrophages and plasma cells; and CD3 (+), CD20 (+), and CD30 (-) in lymphoid cells. In addition, special stains for fungi were negative. Genetic profiling did not detect mutations in V600E, the TERT promoter, H3F3A/HIST1H3B, or IDH1/2. Subsequently, “High-throughput DNA Gene Detection of Pathogenic Microorganisms”, a Next Generation Sequencing (NGS) method, was performed by BGI Genomics [3]. In a total of 25,578,487 reads, 16,376 reads of JC virus were detected at 97.01% coverage (Fig. 1J).

This patient was diagnosed with JC virus-induced progressive multifocal leukoencephalopathy. Mirtazapine (Remeron, MSD) at a daily dose of 15 mg orally was administered, and mefloquine (Lariam, Roche) was administered at a dose of 250 mg weekly one month later. At the same time, Chinese traditional medical herbs, such as *Acorus tatarinowii*, *Poria cocos*, *Rhizoma pinelliae*, and *Scutellaria baicalensis Georgi*, were also given every day. There was a slight improvement in the patient’s condition after one month of treatment, but she remained obtunded and her cognition was severely damaged. After more than two years of postoperative follow-up, her clinical symptoms were still stable and Barthel index scored 35, although there was some progress on radiological imaging (Fig. 1H).

## Discussion and conclusions

Although rare, similar cases of PML in immunocompetent individuals have been reported [1, 2]. PML is regarded as an opportunistic infection secondary to predominantly underlying immunodeficiencies. The recent development of monoclonal antibodies that target the immune system has also been associated with PML [1, 2]. PML may also occur in this patient with minimal or occult immune suppression, which may not be detected clinically. Some elderly patients may also develop PML, presumably due to compromised immune responses associated with the process of aging. Some authors believed that severe deficiency of T-cell immunity (cellular immunity) was necessary for reactivation of JCV. But in our case, the T cell subsets in peripheral blood, including CD3, CD4 and CD8, were detected normal. Except for the sensitivity of peripheral blood, because of CD3-positive in pathology sample, other factors outside the disorder of T cells in triggering PML should be considered. This may better explain why JCV induced PML happened in an immunocompetent patient.

The feature of the pathology associated with PML is JCV infection causing the lysis of myelin-producing cells, which are oligodendrocytes in the central nervous system. The infected oligodendrocytes collect viral particles in the nuclei, which give a bizarre appearance and enlarge as much as 2~3-fold [4]. The enlarged and bizarre-shaped nucleus, which was observed by immunohistochemistry in our case, is the typical feature of pathology for the diagnosis of virus infection.

The most common symptom of PML is cognitive disfunction, and the prognosis is very poor. Most patients die within 4–6 months of onset. Although the treatment for PML is challenging, survival in HIV-infected PML patients has increased substantially during the last decade with the use of highly active antiretroviral therapy (HAART). Only 10% of patients with HIV-infected PML survived for more than a year before the introduction of HAART, and recent studies have shown a 50% one-year survival with HAART treatment [1]. However, there is no specific treatment for HIV-negative patients or immunocompetent patients who develop PML.

Mirtazapine, used in this case, is an antidepressive drug and was also reported to be used empirically in some other non‑HIV PML patients [5]. JC virus entry in astroglial cells in vitro is mediated in part through the 5HTA2 receptors. Mirtazapine could restrict viral spreading within the brain by blocking the 5HTA2 receptor and delay the progression of disease. Mefloquine, an antimalarial drug, is another drug with potentially antiviral effects against JCV. It has been proven that mefloquine can inhibit JCV infection in transformed human glial (SVG-A) cells [6]. However, there is no in vivo experiment to prove its efficacy, as there is no suitable animal model for PML. Pembrolizumab, a PD-1 blocker, was reported effective in some PML cases with previous lymphoma or other immune related diseases [7, 8]. Whether pembrolizumab is also effective in immunocompetent patients need more clinical studies [9].

In conclusion, we present a rare case of central nervous system infection by JC virus in a healthy presumably patient. This may increase our understanding of viral infection when facing the coronavirus epidemic in recent years, considering that similar medications were used.

## Data Availability

Not applicable.

## Abbreviations

JCV: JC virus
PML: Progressive multifocal leukoencephalopathy
CSF: Cerebral spinal fluid
MRI: Magnetic resonance imaging
IHC: Immunohistochemistry
HAART: Highly active antiretroviral therapy
